# Climate change, air pollution and extreme events leading to increasing prevalence of allergic respiratory diseases

**DOI:** 10.1186/2049-6958-8-12

**Published:** 2013-02-11

**Authors:** Gennaro D’Amato, Carlos E Baena-Cagnani, Lorenzo Cecchi, Isabella Annesi-Maesano, Carlos Nunes, Ignacio Ansotegui, Maria D’Amato, Gennaro Liccardi, Matteo Sofia, Walter G Canonica

**Affiliations:** 1 Division of Respiratory and Allergic Diseases, HighSpecialityHospital A. Cardarelli, Department of Respiratory Diseases, Napoli, Italy; 2 Pediatric Hospital, Santa Rosa 381, Research Centre for Respiratory Medicine, Faculty of Medicine, Catholic University Córdoba, Córdoba, Argentina; 3 Allergy and ClinicalImmunologySection, Azienda Sanitaria di Prato, Prato, Italy; 4 INSERM, UMR S 707, EPAR, Paris, France; 5 Centro de ImunoAlergologia de Algarve, Porto, Portugal; 6 Department of Allergy and Immunology, Hospital QuirònBizkaiaErandio, Erandio-Bilbao, Spain; 7 Division of Pneumology, University Hospital Dei Colli-Monaldi and Medical School Federico II, Napoli, Italy; 8 DIMI Allergy and Respiratory Diseases, University of Genova, Genova, Italy

**Keywords:** Airways hyper-responsiveness, Bronchial asthma, Climate change and allergy, Environment and respiratory allergy, Pollen allergy, Respiratory allergy, Urban air pollution

## Abstract

The prevalence of asthma and allergic diseases has increased dramatically during the past few decades not only in industrialized countries. Urban air pollution from motor vehicles has been indicated as one of the major risk factors responsible for this increase.

Although genetic factors are important in the development of asthma and allergic diseases, the rising trend can be explained only in changes occurred in the environment. Despite some differences in the air pollution profile and decreasing trends of some key air pollutants, air quality is an important concern for public health in the cities throughout the world.

Due to climate change, air pollution patterns are changing in several urbanized areas of the world, with a significant effect on respiratory health.

The observational evidence indicates that recent regional changes in climate, particularly temperature increases, have already affected a diverse set of physical and biological systems in many parts of the world. Associations between thunderstorms and asthma morbidity in pollinosis subjects have been also identified in multiple locations around the world.

Allergens patterns are also changing in response to climate change and air pollution can modify the allergenic potential of pollens especially in presence of specific weather conditions.

The underlying mechanisms of all these interactions are not well known yet. The consequences on health vary from decreases in lung function to allergic diseases, new onset of diseases, and exacerbation of chronic respiratory diseases.

Factor clouding the issue is that laboratory evaluations do not reflect what happens during natural exposition, when atmospheric pollution mixtures in polluted cities are inhaled. In addition, it is important to recall that an individual’s response to pollution exposure depends on the source and components of air pollution, as well as meteorological conditions. Indeed, some air pollution-related incidents with asthma aggravation do not depend only on the increased production of air pollution, but rather on atmospheric factors that favour the accumulation of air pollutants at ground level.

Considering these aspects governments worldwide and international organizations such as the World Health Organization and the European Union are facing a growing problem of the respiratory effects induced by gaseous and particulate pollutants arising from motor vehicle emissions.

## 

“*He inhaled a breath of humid morning breeze and let in nitrogen, oxygen, argon, xenon & radon, steam, carbon monoxide, nitrogen dioxide, tetra-ethyl lead, benzene, some mould spores, a bacteria fleet, anonymous body hair, a pigeon ectoparasite, anemophilous pollen, a drop of sulphur dioxide flown from a distant factory, and a particle of dust carried by the night sirocco.*

In other words he breathed air of the city”.

(Stefano Benni*: Achille piè veloce.*Italy: Mondadori; 2003*.*)

## Introduction

The massive increase in emissions of air pollutants due to economic and industrial growth in the last century made air quality an environmental problem of first order in a large number of European and North American countries and it is now an emerging problem in other regions of the world [1-44].

Air pollution is convincingly associated with many signs of asthma aggravation (increased bronchial hyper-responsiveness, visits to emergency departments, hospital admissions, increased medication use etc.) [5,12,13,22-25]. Moreover, sensitive techniques to analyze time-series data have shown that there are clear adverse effects on mortality rates from current levels of air pollution [4,6,7].

Furthermore, several air pollutants, in particular carbon dioxide (CO_2_) and ozone (O_3_), are in the list of greenhouse gases which are involved in the global warming [6-8,11-13].

Global earth’s temperature has markedly risen over the last 50 years due to the increase in greenhouse gas emissions, largely from anthropogenic sources. Changes are also occurring in the amount, intensity, frequency, and type of precipitation as well as the increase of extreme events, like heat waves, droughts, floods, thunderstorms and hurricanes and these are a real and daunting problem [5-15].

It is extremely unlikely that global climatic change can be explained without external forcing. As stated in the Working Group I Report of the Intergovernmental Panel on Climate Change (IPCC) “most of the observed increase in globally averaged temperatures since the mid-20^th^ century is very likely due to the observed increase in anthropogenic greenhouse gas concentrations” [6,7] (Table 1).

**Table 1:** Global warming

	
• The global warming average air temperature has increased by 1.0±0.3°F (0.6±0.2°C) since the late 19^th^ century	
• The average surface temperature of the earth is likely to increase by 2 to 11.5°F (1.1-6.4°C) by the end of the 21^st^ century	
• The average rate of warming over each inhabited continent is very likely to be at least twice as large as that experienced during 20^th^ century	

Working Group I Report of the Intergovernmental Panel on Climate Change (IPCC) 2007.

It has been also observed a rapid rise in the number of hot days and severe meteorological events [9,10,44] such as the 2003 and 2012 heat waves when temperatures reached or went beyond 35°C degrees resulting in excess deaths across Europe [9,10]. Moreover, climate scenarios for the next century predict that the warming will be associated with more frequent and more intense heat waves in wide areas of our planet with increased risk of wildfires and desertification. In urban areas the effects are higher since climate change influences outdoor air pollution because the generation and dispersion of air pollution is in strictly correlation with local patterns of temperature, wind and precipitation.

Climate change has led also to water deprivation in certain areas often associated with water degradation which potentially could result in population migration and the effects on health that result from mass population movement.

Atopy and asthma are more prevalent in developed and industrialized countries as compared with undeveloped and less affluent countries, and the effect of migration is age and time-dependent: early age and longer time increase the likelihood of developing atopy and asthma.

Climate changes will affect the acquisition of atopy and respiratory diseases [12,17].

A number of reports on time trends in allergic respiratory diseases and bronchial asthma have shown a substantial increase in prevalence since the early 1960s [1-3,5].

There is also a link between climate changes and air pollution and an individual’s response to air pollution depends on the source and components of the pollution, as well as on climatic agents [8,11-15]. Some air pollution-related episodes of rhinitis and asthma exacerbation are due to climatic factors that favour the accumulation of air pollutants, such as ozone, at ground level. However, the effects of air pollutants on lung function depend on the environmental concentration of the pollutant, the duration of pollutant exposure and the total ventilation of exposed persons.

A factor clouding the issue is that laboratory evaluations do not reflect what happens during natural exposition, when atmospheric pollution mixtures in polluted cities are inhaled. As a consequence, even if it is plausible that ambient air pollution plays a role in the onset and in the increasing frequency of respiratory allergy, it is not easy to show that this happens at public health level. In addition, it is important to recall that an individual’s response to pollution exposure depends on the source and components of air pollution, as well as meteorological conditions. Indeed, some air pollution-related incidents with asthma aggravation do not depend only on the increased production of air pollution but rather on atmospheric factors that favour the accumulation of air pollutants at ground level.

### Greenhouse gas emissions

**Figure 1:**
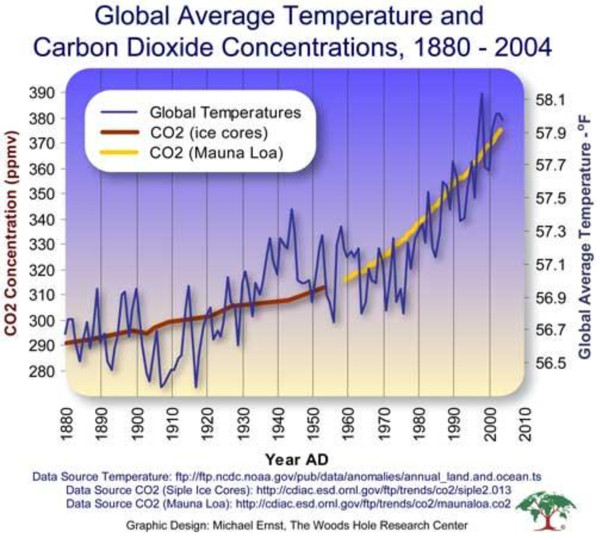
Global average temperature and carbon dioxide concentrations, 1980–2004.

The key determinants of greenhouse gas emissions are energy production, transportation, agriculture and food production and waste management, and attempts at mitigating climate change will need to address each of these. However, while there is some uncertainty about predicting future meteorological trends, whatever interventions may be put in place to ameliorate climate change, it is likely that the world will experience more hot days, fewer frost days, and more periods of heavy rain and consequent flooding [6-8]. Paradoxically it is likely that there will be more periods of drought. A huge increase in CO_2_ concentrations during the last two decades has been experienced (Figure 1). However, it is important to consider that after CO_2_ emissions are reduced and atmospheric concentrations stabilized, surface air temperature continues to rise slowly for a century or more.

### Urban air pollutants

The most abundant components of air pollution in urban areas are nitrogen dioxide, ozone and particulate matter. Sulphur dioxide is particularly abundant in industrial areas. It is estimated that more than 50% of the population of the United States live in areas where levels of ozone, nitrogen dioxide, sulphur dioxide, and particulates exceed current National Ambient Quality Standards, as monitored by the US Environmental Protection Agency [6,7]. With its particulate and gaseous emissions, road traffic is the main contributor to air pollution in most urban areas. Although associations between air pollution and respiratory diseases are complex, recent epidemiological studies have led to an increased recognition of the emerging importance of traffic-related air pollution in both developed and less-developed countries [6,7]. A number of experimental and epidemiological studies confirmed the negative effect of urban air pollution on human health and on allergic respiratory diseases [5,11-17] and projections of climate variability suggest these effects will increase in the next decades [6,7].

#### Nitrogen dioxide (NO_2_)

Car and trucks exhausts, together with power plants, are the most significant sources of outdoor NO_2_, which is a precursor of photochemical smog found in outdoor air in urban and industrial regions and, in conjunction with sunlight and hydrocarbons, results in the production of ozone. Like ozone, NO_2_ is an oxidant pollutant, although it is less chemically reactive. NO_2_ exposure is associated with increased emergency room visits, wheezing, and medication use among children with asthma [20]. Controlled exposure studies of asthmatics have found that NO_2_ can enhance the allergic response to inhaled allergens, and NO_2_ concentrations in ambient air are also reportedly associated with cough, wheezing and shortness of breath in atopic subjects [11-16] A Norwegian study found no association between modeled NO_2_ exposure at the birth address and doctor-diagnosed asthma in a large cohort of children [27].

#### Ozone (O_3_)

Ozone is generated at ground level by photochemical reactions involving nitrogen dioxide, hydrocarbons, and UV radiation. Ozone inhalation induces epithelial damage and consequent inflammatory responses in the upper and lower airways, as shown by an increase in levels of inflammatory cells and mediators in nasal and bronchoalveolar lavage [20].

About 40-60% of inhaled O_3_ is absorbed in the nasal airways, the remainder reaching the lower airways. Exposure to increased atmospheric levels of O_3_ induces reduction of lung function, increased airway hyperreactivity to bronchoconstrictor agents and is related to an increased risk of asthma exacerbations in asthmatic subjects [12-16]. Epidemiologic studies have provided evidence that high ambient concentrations of this air pollutant are associated with an increased rate of asthma exacerbations, increased hospital admissions, and/or emergency department visits for respiratory diseases, including asthma. Furthermore, several studies suggest that O_3_ increases asthma morbidity by enhancing airway inflammation and epithelial permeability [11-16].

O_3_ exposure significantly increases levels of inflammatory cells (in particular neutrophils) and mediators, such as IL-6,IL-8, granulocyte-macrophage colony-stimulating factor (GM-CSF) and fibronectin, in bronchoalveolar lavage fluid (BALF)of asthmatic subjects [16,17].

It has long been speculated that O_3_ and other pollutants may render allergic subjects more susceptible to the antigen they are sensitized [11-13]. It has been observed that the incidence of new diagnoses of asthma is associated with heavy exercise in communities with high concentrations of O_3_, thus, air pollution and outdoor exercise could contribute to the development of asthma [16,17].

However, it is important to take into account that physical exercise in polluted areas results in greater deposition of air pollutants, including allergen-carrying particles, in the lower airways.

The acute health effects of exposure to ambient ozone have been examined in many geographical regions. Potential adverse effects include decrease in lung function, airway inflammation, symptoms of asthma, increases in hospitalization due to respiratory diseases, and excess mortality. Ozone exposure has both a priming effect on allergen-induced responses and an intrinsic inflammatory action in the airways of allergic asthmatics [8,13-16].

In the long term, continuous exposure to high ozone levels impairs respiratory function and causes or exacerbates airway inflammation in healthy subjects and atopic asthmatics. At the population level, long-term exposure to ozone may reduce lung function in school children and adults and increase the prevalence of asthma and asthmatic symptoms [16,17]. In addition, studies have long shown that asthma can be exacerbated by ozone, as measured by increased visits to emergency departments on days with higher levels of ozone and other pollutants [11-13]. Moreover, it has been observed an increased asthma prevalence in milder climatic areas, possibly due to the positive effect of air temperature on ozone synthesis [12-15].

#### Traffic-related air pollution, particulate matter and diesel exhaust particles

There is evidence that living near high-traffic roads is associated with impaired respiratory health including asthma [22-25]. First, McConnell et al. [17] observed that the incidence of newly diagnosed asthma in children is associated with physical exercise in areas with high concentrations of ozone and particulate matter. Since then, other prospective cohort studies have indicated that long-term exposure to traffic pollution could contribute to the development of asthma-like symptoms and allergic sensitization in children [24,25]. Potential long-term effects of traffic exhaust on the development of allergic sensitization were only assessed in the four European birth cohorts.

Long-term exposure to outdoor air pollutants had no association with sensitization in ten-year-old schoolchildren in Norway [27]. These studies, however, are flawed by the fact that no objective assessment of air pollution concentrations was available. Individual exposure was estimated by the distance from the highways.

Particulate matter is a mixture of organic and inorganic solid and liquid particles of different origins, size, and composition It is a major component of urban air pollution and has the greatest effect on health. Penetration of the tracheobronchial area is related to particle size and the efficiency of airway defence mechanisms. Ultrafine particulate matter (UFP), with diameters of 0.1 μm or less, is a major component of emissions from vehicles. These particles accumulate into larger fine PM with a diameter of ≤2.5 mm (PM_2.5_), within short distances from the point of release). PM_10_ (particulate matter with a diameter of 10 mm or less) consists of PM_2.5_ and larger particles of mainly crustal or biological origin, including many aeroallergens. On the basis of epidemiological and laboratory studies, PM_2.5_ appears to be more potent agent for the development of respiratory and cardiovascular disease compared with PM_10_. [21-23] PM_10_ can penetrate the lower airways, and PM_2.5_, is thought to constitute a notable health risk since it can be inhaled more deeply into the lungs at the alveoli level). While human lung parenchyma retains PM_2.5_, particles larger than 5 μm and smaller than 10 μmreach the proximal airways only, where they are eliminated by mucociliary clearance if the airway mucosa is intact [11-13].

A large portion of urban particulate matter originates from diesel engines, the source of diesel exhaust particles (DEPs) which includes other components such as polycyclic aromatic hydrocarbons [PAH]. DEPs account for up to 90% of airborne particulate matter in the world’s largest cities and are composed of fine particles (2.5-0.1 μm) and ultrafine (0.1 μm) particles, which can also coalesce to form aggregates of varying sizes [21].

PM_10_ levels have been associated with early respiratory exacerbations in children with persistent asthma and with higher prevalence rates even after having considered the dispersion of the particles . Although there is compelling evidence that ambient air pollution exacerbates existing asthma, the link with the development of the asthma syndrome is still less well established, as few studies provide extensive exposure data. Researches have elucidated the mechanisms whereby fine particles induce adverse effects; they appear to affect the balance between antioxidant pathways and airway inflammation. Gene polymorphisms involved in antioxidant pathways can modify responses to air pollution exposure. Acute exposure to diesel exhaust causes specific effects, like irritation of nose and eyes, headache, lung function abnormalities, respiratory changes, fatigue, and nausea in acute exposure, while chronic exposure is associated with cough, sputum production, and diminished lung function [21-25].

Studies have demonstrated inflammation in the airways of healthy individuals after exposure to diesel exhaust and DEPs, and elevated expression and concentrations of inflammatory mediators have similarly been observed in the respiratory tract after diesel exhaust and DEP exposure [21,25]. Even if an increased sensitivity of asthmatic individuals to the pro-inflammatory effects of DEPs has not been confirmed, some studies showed a bigger effect of exposure to high-traffic roads in asthmatics compared to non asthmatic subjects, also accompanied by increases in levels of biomarkers of neutrophilic inflammation.

### Effect of climate change on allergic respiratory diseases

A body of evidence suggests that major changes involving the atmosphere and the climate, including global warming induced by human activity, have an impact on the biosphere and human environment [6,7].

Current knowledge on the worldwide effects of climate change on respiratory allergic diseases is provided by epidemiological and experimental studies on the relationship between asthma and environmental factors, like meteorological variables, airborne allergens and air pollution. However, we can try to summarize the correlation between climate changes, allergenic plants and pollen distribution [12-15] in the following points:

(i) increase and faster plant growth;

(ii) increase the amount of pollen produced by each plant;

(iii) increase the amount of allergenic proteins contained in pollen,

(iv) increase the start time of plant growth and therefore the start of pollen production and

(v) earlier and longer growing pollen seasons.

Pollen allergy is frequently used to study the interrelationship between air pollution and allergic respiratory diseases (rhinitis and asthma). Epidemiologic studies have demonstrated that urbanization, high levels of vehicle emissions and westernized lifestyle are correlated to an increase in the frequency of pollen-induced respiratory allergy prevalent in people who live in urban areas compared to those who live in rural areas [12-15].

Meteorological factors (temperature, wind speed, humidity, thunderstorms etc.) along with their climatic regimes (warm or cold anomalies and dry or wet periods, etc.), can affect both biological and chemical components of this interaction. In addition, by inducing airway inflammation, air pollution overcomes mucosal barrier priming allergen-induced responses.

Climate changes might induce negative effects on respiratory allergic diseases favouring the increased length and severity of pollen season, the higher occurrence of heavy precipitation events and the increasing frequency of urban air pollution episodes.

Climatic factors (temperature, wind speed, humidity, thunderstorms, etc.) can affect both components (biological and chemical) of this interaction .

However, the relationship between air pollution, pollen exposure and respiratory allergy is based on an individual’s response to air pollution, which depends on the source and components of the pollution, as well as on climatic agents.

### Thunderstorm-related allergic respiratory diseases and bronchial asthma in pollinosis subjects

There are observations that thunderstorms occurring during pollen season can induce severe asthma attacks in pollinosis patients [45-69].

According to current climate change scenarios, there will be an increase in intensity and frequency of heavy rainfall episodes, including thunderstorms, over the next few decades, which can be expected to be associated with an increase in the number and severity of asthma attacks both in adults and in children (Table 2).

**Table 2:** Weather changes with climate change

	
• More extreme weather patterns, such as increase in thunderstorm	
• High number of thunderstorms in spring and summer at the same time as high pollen counts	
• Pollen grain rupture with thunderstorm with higher level of respirable allergens; also increased in zone	
• More asthma outbreaks	
-UK, Australia and Italy	

From [46,50,57,62] mod.

Associations between thunderstorms and asthma morbidity have been identified in multiple locations around the world [45-69]. So called “thunderstorm asthma” is characterized by asthma outbreaks possibly caused by the dispersion of more respirable allergenic particles derived from pollen and spores by osmotic rupture.

The most prominent hypotheses for thunderstorm-related asthma are linked with bioaerosols, and involve the role of rainwater in promoting the release of respirable particulate matter [47,59,63].

After hydratation and rupture by osmotic shock during the beginning of a thunderstorm, pollen grains may release in atmosphere part of their cytoplasmic content, including inhalable, allergen-carrying paucimicronic particles such as starch granules and other cytoplasmic components .

Thunderstorm-related asthma outbreaks have been described in various geographical zones. One of the first observations regarding thunderstorms and asthma outbreaks was provided by Packe and Ayres at the East Birmingham Hospital (Birmingham, UK) on July 6 and 7, 1983 [45]. These authors described a remarkable increase in the number of asthma emergency department admissions during the hours of a thunderstorm. In a 36-h period, 26 asthma cases were treated in the emergency department, compared with a daily average of two or three cases in the days preceding the outbreak.

Another asthma outbreak occurred in London, UK, coinciding with a heavy thunderstorm on June 24, 1994, when a large increase in the number of visits for asthma at the emergency departments of London and the southwest of England was observed. Several patients who were examined, who were not known to be asthmatics or were affected only by seasonal rhinitis, experienced an asthma attack [50-52]. During a 30-h period from 6 p.m. on June 24, 1994, 640 patients with asthma or other airways disease (283 of whom were not known to be asthmatic and 403 were affected only by seasonal rhinitis) attended several emergency departments, nearly 10 times the expected number of 66 patients. In total, 104 patients were admitted (including five to an intensive care unit) (574 patients attributable to the thunderstorm). Not all affected patients attended a hospital and this epidemic was the largest outbreak ever recorded.

Other asthma outbreaks during thunderstorms have been described in Australia. In Melbourne, two large asthma outbreaks (rapid increase in hospital or general practitioner visits for asthma) coincided with thunderstorms [46]. In WaggaWagga, 215 asthmatic subjects attended the local emergency department, 41 of whom required admission to hospital [57].

In south eastern Australia, Marks et al. [59] observed that the incidence of excess hospital attendances for asthma during late spring and summer was strongly linked to the occurrence of thunderstorm outflows and demonstrated that the arrival of a thunderstorm outflow was accompanied by a large increase in the concentration of ruptured pollen grains in ambient air.

Thunderstorm-related asthma was observed in Naples, Italy, on June 3 and 4, 2004 [62,63], when six adults and one child received treatment in emergency departments. One patient was admitted to an intensive care unit for a very severe bronchial obstruction and acute respiratory insufficiency following a sudden thunderstorm. All individuals were outdoors when the thunderstorm struck. In one severe case, a female sensitized only to Parietaria pollen allergen, soon began to show symptoms of intense dyspnoea, which gradually worsened. She was taken to hospital where she was intubated and given high intravenous doses of corticosteroids. She was discharged a few days later. This patient had previously suffered from seasonal asthma but had been asthma-free for the past few years and did not need continuous therapy. None of the other six subjects took anti-allergic and/or anti-asthma drugs regularly. All seven patients were sensitized with allergic respiratory symptoms upon exposure to Parietaria pollen, but were not sensitized to grasses. Parietaria is an Urticacea that is widespread in the Naples area of Italy with a spring and summer pollen season that is, in part, coexistent with that of grasses. During the thunderstorm, the concentration of airborne Parietaria pollen grains was particularly high, with a peak of 144 grains/m^3^ being recorded on June 3, 2004 [62,63]. Air pollution levels for both gaseous and particulate components based on the hourly concentrations of nitric dioxide, ozone and respirable particulate matter were not particularly high in Naples on June 3 and 4, 2004. Subjects with sensitization to Parietaria who were indoors in Naples with the windows closed during the night between June 3 and 4, 2004, did not experience asthma attacks. No moulds or viruses were involved in the Naples epidemics. Recently, Losappio et al. [69] observed 20 patients with allergic sensitization to Olea pollen who presented to an emergency department in Barletta, Italy, for sudden and severe asthmatic symptoms in May 2010 following a thunderstorm. A similar phenomenon has been suggested for moulds [65] after the observation of a possible key role of sensitization to Alternaria species in thunderstorm related asthma. To date, among pollens, only grass, Parietaria and olive pollen have been suggested as possible key factors in thunderstorm-related asthma [63,66,69]. However, there is a risk of relapse of thunderstorm-related asthma [44,70].

Although thunderstorm-associated asthma outbreaks are not frequent, it is possible to observe in clinical practice single cases of patients with deterioration of the allergic respiratory symptoms during a thunderstorm (Table 3).

**Table 3:** The evidence about thunderstorm-related epidemics of rhinitis and asthma exacerbations

	
1) The occurrence of epidemics is closely linked to thunderstorm.	
2) The thunderstorm-related epidemics are limited to late spring and summer when there are high levels of airborne pollen grains.	
3) There is a close temporal association between the arrival of the thunderstorm, a major rise in the concentration of pollen grains and the onset of epidemics.	
4) Subjects with pollen allergy, who stay indoors with window closed during thunderstorm, are not involved.	
5) There are not high levels of gaseous and particulate components of air pollution during outbreaks.	
6) There is a major risk for subjects who are not under anti-asthma correct treatment.	

In summary the occurrence of these epidemics is closely linked to thunderstorm and they are limited to late spring and summer when there are high levels of airborne pollen grains. There is a close temporal association between the arrival of the thunderstorm, a major rise in the concentration of pollen grains and the onset of epidemics. As a consequence, subjects affected by pollen allergy should be alert to the danger of being outdoors during a thunderstorm in the pollen season.

## Conclusions

In conclusion, strategies to reduce climate changes and air pollution are political in nature, but citizen and in particular health professionals and societies must raise their voices in the decision process to give strong support for clean policies on both national and international levels [71,72].

## Endnotes

Manuscript from a lecture in XXI World Congress of Asthma (Organized by Interasma Global Asthma Association) held in Quebec City in August 18–21 2012.

## Competing interests

The authors declare that they have no competing interests.
